# Systemic and Mucosal Concentrations of Nine Cytokines Among Individuals with *Neisseria gonorrhoeae* infection in Nairobi Kenya

**DOI:** 10.12688/aasopenres.13351.1

**Published:** 2022-03-16

**Authors:** Anne Maina, Marianne Mureithi, John Kiiru, Gunturu Revathi

**Affiliations:** 1 Microbiology, University of Nairobi, NAIROBI, 00202, Kenya; 2 KEMRI, Nairobi, Kenya; 3 Aga Khan University Hospital, Nairobi, Nairobi, Kenya

**Keywords:** Neisseria gonorrhoeae, mucosal cytokines, systemic cytokines, STIs, Kenya

## Abstract

**Introduction**: The human-restricted sexually transmitted *Neisseria gonorrhoeae* (NG) has been shown to modulate the immune response against it and consequently the cytokines produced. The levels of cytokines in NG infection in the African population have not been well described. We aimed to quantify the systemic and mucosal cytokines in NG infection.

**Methods**: This was a comparative cross-sectional study. Levels of nine cytokines (IL-1β, IL-2, IL-4, 1L-6, 1L-10, 1L-12p70, IL-17A, TNF-α and INF-γ) were measured from plasma and genital samples (urethral swabs in men and cervicovaginal lavage in women) from 61 *Neisseria gonorrhoeae* infected individuals seeking treatment for sexually transmitted infections (STIs) at Casino Health Centre in Nairobi, Kenya. A comparative group of 61 NG-uninfected individuals, seeking treatment at the same facility but with laboratory-confirmed negative *Neisseria gonorrhoeae*, *Chlamydia trachomatis* (CT), *Mycoplasma genitalium* (MG) and *Trichomonas vaginalis*(TV) was also included. The Mann-Whitney U test was used to compare the cytokine levels between NG-infected and uninfected individuals. Data was analyzed using STATA ver. 15.1.

**Results**: Overall, systemic IL-6, TNF-α and IL-10 were elevated while genital IL-10 and TNF-α were lower in NG positive participants. On subgroup analysis by sex, the levels of genital IL-1β and IL-6 and systemic IL-6 were elevated in NG-infected men. None of the genital cytokines were elevated in NG-infected women, while all systemic cytokines, except INF-γ, were elevated in NG-infected women.

**Conclusions**: *Neisseria gonorrhoeae* induced the production of different cytokines in men and women, with men having a pro-inflammatory genital response. These differences should be taken into consideration during development of various interventions e.g. vaccine development.

## Introduction

*Neisseria gonorrhoeae* (NG) is a strict human pathogen ^1, 2^. This monoxenous cycle has resulted in the bacterium’s ability to evade both innate and adaptive immune responses mounted against it. Despite the fact that the immune system mounts a robust inflammatory response against NG, immunological memory that would lead to protective immunity against uncomplicated infections has not been defined ^3^. To overcome the innate immune responses, it is able to prevent phagocytosis by neutrophils ^4, 5^, or, if phagocytosis occurs, to survive inside the phagocytic cells ^5^. Further, it is able to evade the host’s nutritional immunity which ensures its survival in the host ^6^. Not only is it able to evade the immune response but also to modulate it. Modulation of the immune response may involve manipulating the response to that which it is able to overcome or evade ^7^, influencing the subtype of immune response cells produced ^8^ or modulation of cell death pathways ^9^. To modulate the immune response, NG drives the differentiation of naïve CD4 ^+ ^T helper (Th) cells to the Th17 axis while actively suppressing Th1 and Th2 responses ^7^. It also induces the production of interleukin 10 (IL-10). IL-10 suppresses the exacerbated inflammatory response. Eventually, the adaptive immune response may also be suppressed ^10^. Consequently, there is increased production of pro-inflammatory cytokines e.g. IL-1β, IL-6, TNF-α, IL-17A. The result is an influx of neutrophils to the site of infection in symptomatic cases but with no immunological memory ^10^.

This ability of NG to evade and modulate the protective response from its human host is happening against a backdrop of increasing reports of antimicrobial resistant (AMR) isolates from around the world ^11– 14^. The current recommended treatment for NG is an extended spectrum cephalosporin (cefixime or ceftriaxone) in combination with azithromycin ^15, 16^. However, cases of both multi-drug resistant (MDR) and extensively drug resistant (XDR) strains have been reported and continue to be reported around the world ^17^. New strategies to combat NG infection are therefore urgently needed. Consequently, NG has been placed in the global high priority pathogen list requiring research and development efforts for new antimicrobial agents ^18^. In addition, the WHO, has set a target of 90% reduction in incident cases of NG by 2030 ^19^, which, although has low mortality, causes significant complications.

The complications of NG infection are of great public health concern. Some of the complications in women include pelvic inflammatory disease (PID) which is a chronic debilitating infection; ectopic pregnancy, rupture of which causes haemorrhage which is the leading cause of maternal mortality; and tubal factor infertility which is one of the commonest causes of infertility ^20^. Complications in men include infertility while in neonates it causes ophthalmia neonatorum ^21^ which can lead to blindness if not treated early. In addition, NG has been associated with the risk of acquisition of other infections such as HIV-1 ^22^. Other potentially fatal conditions which have been described include endocarditis and sepsis ^23, 24^.

Strategies that can be used to combat antimicrobial resistant pathogens include vaccination ^25^ and use of new therapeutics. However the search for an NG vaccine has so far been disappointing ^26^. Knowledge of the cytokine profile in NG infection in different populations will be useful in informing future strategies. The purpose of this study was to determine the systemic and mucosal concentrations of nine cytokines among individuals seeking treatment for sexually transmitted infections in Nairobi, Kenya.

## Methods

### Study setting

Between April and June 2019, we conducted a cross-sectional comparative study of consecutive adults, 18 to 49 years, seeking treatment for sexually transmitted infections at Casino Special Treatment Centre (STC) Health Centre in Nairobi, Kenya. Patients were included if they were between 18 and 49 years and provided written informed consent to participate in the study. Pregnant women were excluded from the study because of the invasive nature of specimen collection (use of specula). Menstruating women were also excluded because the protein-rich menstrual blood has been shown to interfere with mucosal cytokine levels ^27^.

### Sociodemographic data collection

An interviewer-administered questionnaire was used to collect the sociodemographic characteristics and the sexual history. It assessed age, marital status, level of education, occupation, monthly income, circumcision status in men, menarche, sexual orientation, sexual partners in the preceding one year and new sexual partners in the preceding three months. The reported symptoms assessed were discharge and dysuria in men; and discharge, dysuria, vaginal itch, intermenstrual bleeding, post-coital bleeding and lower abdominal pain (LAP) in women.

### Genital sample collection

Vaginal samples were collected after insertion of a speculum. For detection of *Neisseria gonorrhoeae* infection, a dacron swab was placed approximately 2cm into the os and rotated three times, removed and placed into an empty tube and immediately placed on ice. Cervicovaginal lavage (CVL) was collected for mucosal cytokines. Briefly, the cervix and the lateral vaginal walls were flushed with 10 ml normal saline at room temperature. The fluid was allowed to pool into the posterior fornix and aspirated into the same syringe. This procedure was repeated 3-5 times with the same fluid. The fluid was then collected and placed in a 15ml falcon tube and immediately placed on ice to await transportation, up to four hours.

Urethral samples were collected by gently inserting dacron swabs 2–4 cm inside the urethra and rotating three times. They were then carefully removed and placed (i) into an empty tube for detection of *Neisseria gonorrhoeae* infection and (ii) in a tube containing 1.5ml phosphate buffered saline (PBS) for mucosal cytokine analysis. The tubes were immediately placed on ice to await transportation.

For systemic cytokine analysis, 10mls of venous blood was collected, from all participants, into EDTA treated tubes and then immediately placed on ice to await transportation.

All the samples were transported within four hours to the Department of Medical Microbiology, University of Nairobi. Once in the laboratory, the blood was centrifuged at 2000 g for 10 minutes at 4°C After centrifugation, the vacutainers were carefully removed from the centrifuge making sure that the tubes were not inverted, to avoid remixing of the plasma with the cells. Using a transfer pipette, the supernatant was carefully aliquoted into 2ml cryovials and immediately stored at -70°C to await analysis. Cells within the cervicovaginal lavage fluid were separated by centrifugation at 1000 rpm for 10 minutes at 4°C. The supernatant was then stored in 2 ml aliquots at -70°C to await analysis.

### Screening for HIV and STIs

All participants were tested for HIV using Determine HIV Rapid Test (Abbott Diagnostics) with confirmatory testing being done with the First Response™ (Premier Medical Corporation Private Ltd., Gujarat, India), according to Kenya’s Ministry of Heath guidelines. Those who tested negative with the first test were considered HIV negative while those who tested positive with the confirmatory test were considered HIV positive and were either enrolled in the Casino H/C HIV comprehensive care clinic (CCC) or referred to a health facility of their choice for follow-up.

Screening for *Neisseria gonorrhoeae*, *Chlamydia trachomatis*, *Trichomonas vaginalis* and *Mycoplasma genitalium* was done using Multiplex PCR (Sacace Biotechnologies **,**Como, Italy) according to the manufacturer’s instructions.

### Measurement of cytokines

The systemic and genital levels of 9 cytokines (IL-1β, IL-2, IL-4, IL-6, IL-10, IL-12p70, IL-17A, TNF-α and IFN-γ) were measured using the Invitrogen™ High Sensitivity 9-Plex Human ProcartaPlex™ Panel (Thermo Fisher Scientific Inc.)

Briefly, samples were first thawed. Fifty μL of diluted magnetic beads were added to each well. The plates were washed and samples and standards added to the wells (25 μL of samples and 25 μL of standards). The plates were then shaken at room temperature for 30 min, after which they were transferred at 4°C in the dark. Shaking for 30 minutes at room temperature was then done and 25 μL of Detection Antibody Mix was added followed by incubation with shaking at room temperature for 30 min in the dark. The beads were then washed twice, 50 μL of Streptavidin-PE was added and the beads washed twice again. Incubation with shaking at room temperature for 30 min in the dark was done followed by washing twice. 50 μL of Amplification Reagent 1 and 2 were added with shaking at room temperature for 30 min in the dark. 50 μL of Amplification Reagent 2 was added followed by incubation with shaking at room temperature for 30 min in the dark. The beads were then washed twice. Reading buffer (120 μL) was added and shaking at room temperature for 5 min in the dark done. Data was acquired on the Magpix™ system.

The lower limits of detection were: IFN-γ (1.511 pg/ml), TNF-α (0.859 pg/ml), IL-1β (0.199pg/ml), IL-2 (0.571 pg/ml), IL-4 (0.999 pg/ml), IL-6 (1.047 pg/ml), IL-10 (0.17pg/ml), IL-17A (0.227 pg/ml), IL-12p70(0.615pg/ml).

### Statistical analysis

Data was entered into RedCap ^28^ hosted at APHRC and analysed using STATA version 15.1. Categorical variables were compared using the χ2 or Fisher’s exact test. Cytokine levels were compared using the Mann-Whitney U test or the Student t test after tests of normality using the Shapiro-Wilk test. The level of statistical significant was set at <0.05.

### Ethical approval

The study received ethical approval from the Kenyatta National Hospital/University of Nairobi (KNH/UoN) ERC (Protocol number (P304/06/2017). Permission to conduct the study at Casino H/C was granted by Nairobi County’s Department of Health Services.

## Results

### Demographic characteristics

Between April and June 2019, a total of 297 individuals, 18 to 49 years, were recruited. Of these, 122 were included in this comparative cross-sectional study (61 NG positive, and 61 NG negative). Co-infections with three organisms: *Chlamydia trachomatis*(CT), *Mycoplasma genitalium*(MG) and *Trichomonas vaginalis*(TV) were excluded from those in the NG infection group; while infections with four organisms: NG, CT, TV and MG were excluded from those in the NG negative group. MS Excel random number generator was used to select participants in the NG negative group.

Table 1 summarizes the sociodemographic characteristics, reported symptoms at recruitment and sexual history of the study participants. There was a significant difference between infected and uninfected participants with regards to sex composition (p=<0.001). In addition, there was a significant difference between groups in participants reporting dysuria, number of sexual partners and having a new sexual partner in the previous one year.

**Table 1.** Sociodemographic characteristics of the study participants.

Characteristic	NG-positive(n=61)		NG-negative (n=61)		p-value
	n	%	n	%	
Age (median, IQR) (n=122)	30	(26-36)	28	(23-35)	0.09
**Age category**					0.014
<25 years	10	16.39	22	45.90	
≥ 25 years	51	83.61	39	63.93	
**Sex**					<0.001
Male	45	73.77	21	34.43	
Female	16	26.23	40	65.57	
**Sexual debut (median, IQR)**	18	(16-20)	19	(17-20)	0.359
**Sexual debut**					0.427
< 18 years	20	32.79	16	26.23	
≥ 18 years	41	67.21	45	73.77	
**Circumcision status ^†^**					0.159
Yes	43	95.56	18	85.71	
No	2	4.44	3	14.29	
**Self-reported symptoms at recruitment**					
**Any symptom**					
Yes					
No					
**Discharge**					0.362
Yes	51	83.61	47	77.05	
No	10	16.39	14	22.95	
**Dysuria**					0.004
Yes	47	77.05	32	52.46	
No	14	22.95	29	47.54	
**LAP ^±^**					0.587
Yes	10	62.50	28	70.00	
No	6	37.50	12	30.00	
**Vaginal itch ^±^**					0.007
Yes	9	56.25	22	55.00	
No	7	43.75	18	45.00	
**Intermenstrual bleeding ^±^**					0.219
Yes	3	18.75	3	7.50	
No	13	81.25	37	92.50	
**Postcoital bleeding ^±^**					0.111
Yes	1	6.25	0	0	
No	15	93.75	40	100.00	
**Sexual partners last 1 year**					0.009
1	22	36.07	38	62.30	
2	25	40.98	13	21.31	
3	9	56.25	4	6.56	
4	0	0	3	4.92	
>4	5	8.20	3	4.92	
**New sexual partner in last 3 months**					0.001
Yes	32	52.46	14	22.95	
No	29	47.54	47	77.05	
**HIV infection**					0.309
Positive	3	4.92	1	1.64	
Negative	58	95.08	60	98.36	

^±^Female participants only ^†^Male participants only

### Concentration of cytokines

Table 2 shows the concentration of cytokines in the two groups. In the systemic cytokines, more than half of the study participants in both groups had undetectable levels of IL-10 and TNF-α. The NG positive participants had higher concentrations of IL-6, IL-10 and TNF-α ( *p*= 0.028, 0.052 and 0.012 respectively).

**Table 2.** Cytokine levels in the study participants.

Cytokine (pg/ml)	NG-positive (n=61)				NG-negative(n=61)				P-value
	Detectable				Detectable				
	n	(%)	median	(IQR)	n	%	median	(IQR)	
**Systemic cytokines**									
IL-1β	61	(100.00)	0.77	(0.38-4.16)	60	(98.36)	0.76	(0.35-1.46)	0.243
IL-2	57	(93.44)	3.05	(1.61-16.04)	54	(88.52)	3.36	(2.02-5.35)	0.750
IL-4	40	(65.57)	1.88	(0.82-11.27)	36	(59.02)	3.19	(1.36-6.13)	0.185
IL-6	56	(91.80)	5.85	(2.82-24.57)	53	(86.89)	4.51	(2.17-6.65)	0.028
IL-10	18	(29.51)	0.46	(0.18-3.06)	20	(32.79)	0.20	(0.09-0.66)	0.052
IL-12p70	56	(91.80)	0.75	(0.52-2.001)	49	(80.33)	0.86	(0.55-2.15)	0.644
Il-17A	60	(98.36)	0.78	(0.45-3.92)	55	(90.16)	0.75	(0.41-1.47)	0.683
IFN-γ	46	(75.41)	0.60	(0.42-0.98)	25	(40.98)	0.53	(0.43-0.79)	0.576
TNF-α	25	(40.98)	11.89	(2.67-27.68)	22	(36.07)	1.66	(0.96-13.13)	0.012
**Genital cytokines**									
IL-1β	60	(98.36)	100.34	(8.33-583.27)	56	(91.80)	43.22	(1.13-352.64)	0.230
1L-2	15	(24.59)	6.163	(0.88-14.53)	24	(39.34)	10.067	(7.98-12.88)	0.505
1L-4	2	(3.28)	2.0985	(1.23-2.97)	2	(3.28)	20.16	(19.24-21.08)	0.121
IL-6	41	(67.21)	59.891	(20.69-125.07)	40	(65.57)	87.82	(42.65-239.66)	0.081
IL-10	35	(57.38)	0.418	(0.20-2.41)	27	(44.26)	2.62	(1.71-5.85)	0.001
IL-12p70	12	(19.67)	0.186	(0.17-0.20)	8	(13.11)	0.225	(0.18-5.64)	0.174
IL-17A	40	(65.57)	2.744	(0.9-8.00)	37	(60.66)	4.211	(2.42-13.22)	0.079
IFN-γ	26	(42.62)	0.728	(0.28-4.29)	13	(21.31)	28.994	(0.30-65.45)	0.173
TNF-α	40	(65.57)	11.37	(3.03-23.60)	39	(63.93)	25.119	(15.96-34.60)	0.001

In the genital cytokines, more than half of the participants had undetectable levels of IL-2, IL12p70, and IFN-γ. Further, only 4 participants had detectable IL-4 levels. The levels of IL-10 and TNF-α were statistically significantly lower in NG positive individuals than in NG negative individuals (both *p*=0.001)

### Subgroup analysis of cytokine concentration by sex

We proceeded to carry out subgroup analysis of cytokine concentration by sex since the sex composition was statistically significantly different in both groups ( *p*=<0.001, Table 1). Table 3 shows the subgroup analysis of cytokine concentrations by sex.

**Table 3.** Sub-group analysis of cytokines by sex.

Female participants									
Cytokine (pg/ml)	NG-positive (n=16)				NG-negative (n=40)				P-value
	Detectable				Detectable				
	n	(%)	median	(IQR)	n	(%)	median	(IQR)	
**Systemic cytokines**									
IL-1β	16	(100.00)	7.67	(6.38-9.93)	39	(97.50)	0.81	(0.35-2.37)	<0.001
IL-2	14	(87.50)	24.41	(19.06-27.27)	34	(85.00)	3.75	(2.27-11.95)	<0.001
IL-4	11	68.75	21.56	(14.56-23.94)	25	(62.50)	4.04	(2.97-12.28)	0.001
IL-6	16	(100.00)	35.11	(26.07-47.60)	36	(90.00)	5.0	(3.59-10.73)	<0.001
IL-10	6	(37.5)	6.84	(3.06-13.19)	19	(47.50)	0.22	(0.09-0.74)	0.002
IL-12p70	12	(75.00)	16.01	(12.95-18.85)	29	(72.50)	1.59	(0.83-13.15)	0.003
IL-17A	16	(100.00)	6.71	(5.16-8.58)	36	(90.00)	0.88	(0.61-3.61)	<0.001
IFN-γ	3	(18.75)	19.71	(18.93-24.67)	5	(12.50)	1.86	(1.57-2.37)	0.180
TNF-α	12	(75.00)	30.04	(22.24-41.56)	16	(40.00)	1.41	(0.96-15.48)	<0.001
**Genital cytokines**									
IL-1β	15	(93.75)	152.27	(19.68-1375.17)	39	(97.50)	100.42	(30.09-960.61)	0.946
IL-2	8	(50.00)	14.53	(13.60-16.33)	19	(47.50)	11.51	(7.98-17.95)	0.251
IL-4	0	(0)	-	-	2	(5.00)	20.16	(19.24-21.08)	-
IL-6	13	(81.25)	125.07	(62.21-494.17)	37	(92.50)	89.60	(51.67-304.10)	0.588
IL-10	8	(50.00)	3.27	(2.57-4.80)	22	(55.00)	4.15	(2.30-6.65)	0.981
IL-12p70	0	(0)	-	-	2	(5.00)	18.33	(10.99-25.66)	-
Il-17A	10	(62.50)	16.36	(3.21-20.62)	33	(82.50)	5.00	(2.42-13.22)	0.300
INF-γ	3	(18.75)	72.58	(61.85-103.22)	7	(17.50)	65.45	(46.42-213.52)	0.569
TNF-α	14	(87.50)	25.88	(15.96-58.31)	36	(90.00)	25.12	(18.54-34.6)	0.914
Male Participants									
Cytokine (pg/ml)	NG-positive (n=45)				NG-negative (n=21)				P-value
	n	(%)	median	(IQR)	n	(%)	median	(IQR)	
**Systemic cytokines**									
IL-1β	45	(100.00)	0.59	(0.34-0.98)	21	(100.00)	0.53	(0.34-0.95)	0.746
IL-2	43	(70.49)	2.39	(1.46-3.64)	20	(95.24)	2.56	(1.61-3.74)	0.971
IL-4	29	(47.54)	0.95	(0.74-2.41)	11	(52.38)	1.23	(0.51-2.17)	0.868
IL-6	40	(65.57)	3.97	(1.88-6.92)	17	(80.95)	2.40	(1.29-3.37)	0.010
IL-10	12	(26.67)	0.33	(0.14-0.46)	1	(4.76)			0.109
IL-12p70	44	(97.78)	0.65	(0.45-0.96)	20	(95.24)	0.62	(0.45-0.82)	0.510
Il-17A	44	(97.78)	0.50	(0.28-0.93)	19	(90.48)	0.45	(0.25-0.82)	0.515
INF-γ	43	(95.56)	0.56	(0.42-0.92)	20	(95.24)	0.50	(0.40-0.64)	0.207
TNF-α	13	(28.89)	2.67	(1.00-4.08)	6	(28.57)	1.68	(0.77-2.67)	0.481
**Genital cytokines**									
IL-1β	45	(100.00)	93.66	(2.78-542.93)	17	(80.95)	0.4	(0.18-1.11)	<0.001
IL-2	7	(15.56)	0.88	(0.20-1.52)	5	(23.81)	0.20	(0.20-0.96)	0.728
IL-4	2	(4.44)	2.10	(1.23-2.97)	0	(0)			
IL-6	28	(62.22)	37.20	(15.41-87.39)	3	(14.29)	8.31	(5.09-17.17)	0.038
IL-10	27	(60.00)	0.28	(0.12-0.60)	5	(23.81)	0.05	(0.02-0.26)	0.113
IL-12p70	12	(26.67)	0.19	(0.17-0.20)	6	(28.57)	0.19	(0.17-0.26)	0.623
Il-17A	30	(66.67)	2.24	(0.84-4.61)	4	(19.05)	1.16	(0.21-8.70)	0.487
INF-γ	23	(51.11)	0.41	(0.28-3.13)	6	(28.57)	0.29	(0.28-0.34)	0.201
TNF-α	26	(57.78)	4.16	(2.45-11.69)	3	(14.29)	1.79	(1.72-3.32)	0.185

In female participants, systemic IL-10 and IFN-γ were detectable in less than half of the participants. Genital IL-4 and IL-12p70 were not detectable in NG positive participants.

All systemic cytokines except IFN-γ were present in higher concentrations in NG infected women than in NG negative women. However, there were no statistically significant differences in the genital cytokine concentrations in the nine analysed cytokines ( Table 3).

In male participants, systemic IL-10 and TNF-α were undetectable in more than half of the NG positive participants. Genital IL-4 was detectable in only two participants. On the other hand, systemic IL-1β was detectable in all the participants. 

In NG negative male participants, systemic IL-1β was detectable in all participants while only one participant had detectable levels of IL-10. In genital cytokines, only IL-1β was detectable in more than half of the participants with other cytokines being detected in none or only a few participants : ranging from 0/21; 0% (IL-4) to 6/21; 28.67%(IL-12p70 and IFN-γ).

The levels of systemic and genital IL-6 and genital IL-1β were statistically significantly higher in NG positive men compared to NG negative men ( *p*=0.010, 0.038 and <0.001 respectively).There were no significant differences in the other cytokines ( Table 3).

## Discussion

In this study, we assessed the genital and systemic levels of nine cytokines: IL-1β, IL-2, IL-4 IL-6, IL-10, IL-12p70, IL-17A, IFN-γ and TNF-α in *Neisseria gonorrhoeae* infected individuals. Overall, the levels of systemic IL-6, TNF-α and IL-10 were elevated while genital IL-10 and TNF-α were lower in NG positive participants. By contrast, some studies have shown that NG induces an anti-inflammatory state with increased production of IL-10 in the genital tract ^10, 29^ while another study showed increased TNF-α production leading to protection of NG-infected cells from undergoing apoptosis ^30^. However, with evidence showing that NG uses different mechanisms to establish infection in the male and female genital tracts ^31^, and that the immune response is likely to be gender specific, the cytokine profile in different gender should be considered differently.

In our study, NG-infected men had elevated genital IL-1β and IL-6 as well as elevated systemic IL-6. This is similar to findings by Ramsey *et al.* (1995) of sequential increase in IL-6 in urine before the onset of symptoms and of IL- 1β at the onset of symptoms in some of the participants ^32^. Expression of genital IL-1β has been associated with inflammation and symptomatic infection and was significant in disease progression ^33^. IL-6 production is important in clearance of infection and restoration of damaged tissue ^34^.

In women, none of the assayed genital cytokines was elevated. This is similar to findings by Hedges *et al.* (1998) and Cauci & Culhane (2007), showing that NG had no effect on genital cytokines ^35, 36^. In contrast, NG was associated with elevated genital cytokines (IL-1β, IL-12p70, TNF-α, IL-2, and IL-17 among others) in a study involving HIV-infected and uninfected women in South Africa ^37, 38^. The lack of upregulation of any of the assayed cytokines could be explained by the fact that NG has been shown to modulate the immune response and suppress proinflammatory responses ^39^. On the other hand, eight out of the nine assayed systemic cytokines were elevated in NG-positive women. 

Immune responses at the mucosal level have been associated with systemic responses. Indeed, Morrison *et al.* (2020) argue that the imbalances of systemic and genital cytokines may explain the increased risk of acquisition of some infections e.g. HIV ^40^ and therefore stressing the need of considering both responses at the mucosal and the systemic level.

Our study had several limitations. First, we did not screen for bacterial vaginosis (BV). BV has been associated with a pro-inflammatory response in the genital tract and is prevalent in the African population ^41^. Secondly, we did not quantify the hormonal levels in women. Progesterone has been associated with inhibition of inflammatory effects of NG infection and a reduction of genital levels of IL-1β, TNF-α, and IL-6 with ^42^ resultant asymptomatic infection. Future studies should explore the relationship between cytokine and progesterone levels. Thirdly, the microenvironments evaluated are quite heterogenous. This has the potential to interfere with the significance and interpretation of the results obtained. Lastly, we used the lower limits of detection set by the manufacturer. Given that polypeptides are synthesized and secreted at different intensities depending on the microenvironment evaluated, there is the potential for experimental bias. Our results can therefore not be generalized to the general population.

Despite the limitations, our study quantifies nine cytokine profile in NG and shows differences in men and women. Future studies can explore these differences using a larger sample size and controlling for possible confounders such as progesterone levels and bacterial vaginosis.

## Data availability

Mendeley Data. Systemic and mucosal concentrations of nine cytokines among individuals with Neisseria gonorrhoeae infection in Nairobi, Kenya. DOI: 10.17632/67b55dncm7.1 ^43^

This project contains the following underlying data:

- We conducted a cross-sectional comparative study of consecutive adults, 18 to 49 years, seeking treatment for sexually transmitted infections at Casino Special Treatment Centre (STC) Health Centre in Nairobi, Kenya.
- Patients were included if they were between 18 and 49 years and provided written informed consent to participate in the study. An interviewer-administered questionnaire was used to collect the sociodemographic characteristics and the sexual history. Vaginal samples were collected after insertion of a speculum. For detection of Neisseria gonorrhoeae infection, a dacron swab was placed approximately 2cm into the os and rotated three times, removed and placed into an empty tube and immediately placed on ice. Cervicovaginal lavage (CVL) was collected for mucosal cytokines.
- Briefly, the cervix and the lateral vaginal walls were flushed with 10 ml normal saline at room temperature. The fluid was allowed to pool into the posterior fornix and aspirated into the same syringe. This procedure was repeated 3-5 times with the same fluid.
- The fluid was then collected and placed in a 15ml falcon tube and immediately placed on ice to await transportation, up to 4 hours. Urethral samples were collected by gently inserting dacron swabs 2-4 cm inside the urethra and rotating three times. They were then carefully removed and placed (i) into an empty tube for detection of Neisseria gonorrhoeae infection and (ii) in a tube containing 1.5ml phosphate buffered saline (PBS) for mucosal cytokine analysis. The tubes were immediately placed on ice to await transportation.
- For systemic cytokine analysis, 10mls of venous blood was collected, from all participants, into EDTA treated tubes and then immediately placed on ice to await transportation. All participants were tested for HIV using Determine HIV Rapid Test (Abbott Diagnostics) with confirmatory testing being done with the First Response™ (Premier Medical Corporation Private Ltd., Gujarat, India), according to Kenya’s Ministry of Heath guidelines.
- Those who tested negative with the first test were considered HIV negative while those who tested positive with the confirmatory test were considered HIV positive and were either enrolled in the Casino H/C HIV comprehensive care clinic (CCC) or referred to a health facility of their choice for follow-up. Screening for Neisseria gonorrhoeae, Chlamydia trachomatis, Trichomonas vaginalis and Mycoplasma genitalium was done using Multiplex PCR (Sacace Biotechnologies, Como, Italy) according to the manufacturer’s instructions.
- The systemic and mucosal levels of 9 cytokines (IL-1β, IL-2, IL-4, IL-6, IL-10, IL-12p70, IL-17A, TNF-α and IFN-γ) were measured using the Invitrogen™ High Sensitivity 9-Plex Human ProcartaPlex™ Panel (Thermo Fisher Scientific Inc.)
