# Supplementary material for: Systemic and Mucosal Concentrations of Nine Cytokines Among Individuals with Neisseria gonorrhoeae infection in Nairobi Kenya
Source: AAS Open Res. 2023 Oct 31;5:12. Originally published 2022 Mar 16. [Version 2] doi: 10.12688/aasopenres.13351.2 (PMC10839855; doi:10.12688/aasopenres.13351.2)
Supplement: Peer review history

# Reviewer report: Reviewer response for version 2

**DOI:** 10.21956/aasopenres.15647.r30460

**Published:** 2023-12-29

**Authors:** Qian Yu

**Affiliations:** 1 Yale School of Medicine, New Haven, Connecticut, USA

The current study tested the systemic and vaginal levels of nine cytokines within the NG-positive and negative populations. The cytokine levels between female and male, the positive and negative groups were analyzed.

 In the Sociodemographic data collection session, a variety of information was collected including sexual history etc.. If the information is available, I am interested to know about the prior history of vaginal infection, including NG and other STI. As far as NG infection in males, it has been shown that repeated infection can occur even after antibiotic treatment. The prior infection can potentially affect the cytokine levels that tested in the current study. Thus, it should be considered when you recruited the participants and interpreted your date. 

 NG infection in women is far more complicated than in men. Majority of early infection in women is asymptomatic. That is probably why you only had 16 NG-positive females recruited. It is a small sample size. In the Sociodemographic data collection session, the lower abdominal pain was mentioned. If it was present in some of your participants, it indicated the dissemination of NG infection. I think the female infection should be discussed in more detail. 

 In Table 1, I noticed that a portion of NG-negative participants displayed self-reported symptoms including discharge (47 out of 61), dysuria (32 out of 61), LAP and vaginal itch. You excluded the infection with NG and three other organisms. Please discuss the possible reasons for the above symptoms and the potential impact of the symptoms on the cytokine levels.

Is the work clearly and accurately presented and does it cite the current literature?

Yes

If applicable, is the statistical analysis and its interpretation appropriate?

Yes

Are all the source data underlying the results available to ensure full reproducibility?

Yes

Is the study design appropriate and is the work technically sound?

Yes

Are the conclusions drawn adequately supported by the results?

Partly

Are sufficient details of methods and analysis provided to allow replication by others?

Yes

Reviewer Expertise:

Microbiology, Neisseria gonorrhoeae, Cell biology

I confirm that I have read this submission and believe that I have an appropriate level of expertise to confirm that it is of an acceptable scientific standard, however I have significant reservations, as outlined above.

# Reviewer report: Reviewer response for version 2

**DOI:** 10.21956/aasopenres.15647.r30357

**Published:** 2023-12-28

**Authors:** Won Fen Wong

**Affiliations:** 1 Department of Medical Microbiology, Universiti Malaya, Kuala Lumpur, Federal Territory of Kuala Lumpur, Malaysia

The current version of article is satisfactory.

Is the work clearly and accurately presented and does it cite the current literature?

Yes

If applicable, is the statistical analysis and its interpretation appropriate?

Yes

Are all the source data underlying the results available to ensure full reproducibility?

Yes

Is the study design appropriate and is the work technically sound?

Yes

Are the conclusions drawn adequately supported by the results?

Partly

Are sufficient details of methods and analysis provided to allow replication by others?

Yes

Reviewer Expertise:

Immunology

I confirm that I have read this submission and believe that I have an appropriate level of expertise to confirm that it is of an acceptable scientific standard.

# Reviewer report: Reviewer response for version 2

**DOI:** 10.21956/aasopenres.15647.r30356

**Published:** 2023-11-14

**Authors:** Charles D. Kato

**Affiliations:** 1 School of Bio-Security, Biotechnical & Laboratory Sciences, College of Veterinary Medicine, Animal Resources & Bio-Security, Makerere University, Kampala, Uganda

The authors report systemic and mucosal cytokine profiles associated with Neisseria gonorrhoeae. The proposed work has implications in elucidating the observed disease spectrum. I am now happy with the current version of the manuscript and I would recommend it for indexing.

Is the work clearly and accurately presented and does it cite the current literature?

Partly

If applicable, is the statistical analysis and its interpretation appropriate?

Partly

Are all the source data underlying the results available to ensure full reproducibility?

Partly

Is the study design appropriate and is the work technically sound?

Yes

Are the conclusions drawn adequately supported by the results?

Partly

Are sufficient details of methods and analysis provided to allow replication by others?

Partly

Reviewer Expertise:

Clinical Immunology and population genetics

I confirm that I have read this submission and believe that I have an appropriate level of expertise to confirm that it is of an acceptable scientific standard.

# Reviewer report: Reviewer response for version 1

**DOI:** 10.21956/aasopenres.14496.r29669

**Published:** 2023-06-15

**Authors:** Won Fen Wong

**Affiliations:** 1 Department of Medical Microbiology, Universiti Malaya, Kuala Lumpur, Federal Territory of Kuala Lumpur, Malaysia

This study investigated inflammatory cytokines in the systemic and genital components of the Neisseria gonorrhoea infected individuals versus the uninfected controls. 

 The results presented were merely detected or undetected in Table 2. It is not clear what is the median value stated in the Table. It would be good if proper concentrations with unit can be provided.

 A relatively high number of uninfected individuals showed detection of inflammatory cytokines. Have the authors examined if these individuals are also free of other types of infection? Or is it because the detection limit is very low using this highly sensitive kit? It would be good if a dot plot can be provided.

Is the work clearly and accurately presented and does it cite the current literature?

Yes

If applicable, is the statistical analysis and its interpretation appropriate?

Yes

Are all the source data underlying the results available to ensure full reproducibility?

Yes

Is the study design appropriate and is the work technically sound?

Yes

Are the conclusions drawn adequately supported by the results?

Partly

Are sufficient details of methods and analysis provided to allow replication by others?

Yes

Reviewer Expertise:

Immunology

I confirm that I have read this submission and believe that I have an appropriate level of expertise to confirm that it is of an acceptable scientific standard, however I have significant reservations, as outlined above.

# Reviewer report: Reviewer response for version 1

**DOI:** 10.21956/aasopenres.14496.r29171

**Published:** 2022-05-27

**Authors:** Charles D. Kato

**Affiliations:** 1 School of Bio-Security, Biotechnical & Laboratory Sciences, College of Veterinary Medicine, Animal Resources & Bio-Security, Makerere University, Kampala, Uganda

The authors describe a panel of cytokines among Neisseria gonorrhoeae-positive individuals and controls in Nairobi Kenya. Understanding cytokine profiles in a case-control approach is important in elucidating key parameters surrounding the pathogenesis of the disease so as to come up with appropriate interventions. However, the paper has some major concerns that need to be addressed.

- In addition to Neisseria gonorrhoeae, patients were screened for HIV, Chlamydia trachomatis, Trichomonas vaginalis, and Mycoplasma genitalium. It is not clear why only these diseases were considered, an explanation needs to be provided. In the result section, patients with NG, CT, TV, and MG co-infections were excluded from further analysis but not HIV (why so?). I would have loved to see the effect of these co-infections on the cytokine profiles if they indeed had no effect then exclusion would not have been important and this would have increased the power of the study.
- The data analysis section needs to be expanded as it’s not clear why the authors used non-parametric tests. Did the authors just assume that the data was not normally distributed, otherwise, an analysis of normality for the data set should have been done to guide downstream analyses? It would also be helpful to indicate which tests were done for which data set.
- Furthermore, I do not buy the argument of comparing genital cytokine levels between females and males as indicated in table 3. Mucosal NG in both sexes presents differently and such a comparison would not yield. Indeed, even the sample collection between the 2 was from different anatomical structures making comparisons impossible. I would advise to delete this from the table 3 and concentrate on systemic cytokines.
- My thinking is also that this study would have provided more insights into the different clinical spectrum for NG. I would therefore have loved to see how the cytokine profiles would vary across the different clinical presentations as this would have made the work more novel and interesting. For example, in this study, did all males and females present with the same clinical spectrum, and if not do cytokines have role to play? Or are individuals with less severe clinical spectrum associated with a given cytokine profile?
- The discussion section of the paper, needs to be well articulated with more evidence. The implication of elevated systemic cytokines in NG patients needs to be elucidated and what this would mean on disease prognosis.
- The cytokine nomenclature is also not consistent and needs to be clarified. In some cases, IFN-g or TNF-a or b are used. Standard nomenclature for these should be used throughout and consistently throughout the manuscript.
- For all equipment mentioned, the manufacturer and country of origin should be indicated.

Is the work clearly and accurately presented and does it cite the current literature?

Partly

If applicable, is the statistical analysis and its interpretation appropriate?

Partly

Are all the source data underlying the results available to ensure full reproducibility?

Partly

Is the study design appropriate and is the work technically sound?

Yes

Are the conclusions drawn adequately supported by the results?

Partly

Are sufficient details of methods and analysis provided to allow replication by others?

Partly

Reviewer Expertise:

Clinical Immunology and population genetics

I confirm that I have read this submission and believe that I have an appropriate level of expertise to confirm that it is of an acceptable scientific standard, however I have significant reservations, as outlined above.
