# Supplementary material for: Systemic and Mucosal Concentrations of Nine Cytokines Among Individuals with Neisseria gonorrhoeae infection in Nairobi Kenya
Source: AAS Open Res. 2022 Mar 16;5:12. [Version 1] doi: 10.12688/aasopenres.13351.1 (PMC10839855; doi:10.12688/aasopenres.13351.1)
Supplement: Peer review history

# Response

**Published:** 2023-10-25

**Authors:** Anne Maina

**Affiliations:** University of Nairobi, Kenya

Comment 1:  The median value and the IQR in the table indicate the median of the detectable cytokines in the study population. The concentrations are in pg/ml. Undetectable cytokines have not been included in the table.   Comment 2:  The presence of inflammatory cytokines in uninfected individuals could be due to other undetected infections or non-infectious conditions. Alternatively, it could be due to the high sensitivity of the assay capturing normal low-levle immune activity. Future studies could include a wider range of infections especially those endemic in the study area, and which could affect the immune response.

# Response

**Published:** 2023-10-25

**Authors:** Anne Maina

**Affiliations:** University of Nairobi, Kenya

Response to Reviewer 1; comment 1: The rationale of the exclusion of CT, TV and MG co-infections in the analysis, was to isolate the cytokine responsese specific to NG infection. The presence of multiple infections could confound the interpretation of the results, as each pathogen could, potentially, modulate the immune response in a different manner. We did not exclude HIV co-infections because of their chronic nature and the high prevalence of HIV-NG co-infections, which we believe reflects a real-world scenario. In future research, it would be important to include a separate analysis that considers the impact of co-infections. This would provide a more comprehensive understanding of the immune responses in the context of STIs and could potentially inform the development of more effective treatment strategies.   Comment 2: The Shapiro-Wilk test was used to test for normality. This has now been clarified in the materials and methods section. Comment 3 response: The differing pathogenesis and anatomical structures in men and women is indeed a valid point. In Table 3, we have presented the results disaggregated by sex separately but within the same table for ease of reference.    Comment 4 response We note the importance of providing more insights into the clinical spectra of NG. Exploring these differences could provide further insights into the immunological mechanisms underlying these diverse clinical outcomes. In our current study, we focused on the overall cytokine response to NG infection, without stratifying by clinical presentation. However, we agree that your suggestions could add significant value to our work. The fact that the symptoms were self-reported and therefore subjective, could not allow us to stratify the symptoms using these self-reported symptoms. For future studies, we will consider investigating the association between cytokine profiles and various clinical presentations, such as asymptomatic versus symptomatic infections, mild versus severe cases, and uncomplicated versus complicated infections.   Comment 5 response Elevated systemic cytokines in NG patients suggest a significant immune response. The increased levels of pro-inflammatory cytokines like IL-6 and TNF-alpha indicate an inflammatory response while elevated systemic IL-10, an anti-inflammatory cytokine could suggest a regulatory response to control inflammation. These changes might impact disease progression and could potentially contribute to complications associated with NG infection. Moreover, these cytokine profiles could potentially serve as biomarkers for different disease stages and in the design of new therapeutic strategies.   Comment 6 response: Stadanrd nomencalture for the cytokines has been used consisstently througyout the text.   Comment 7 response:  The manufacturer and county of origin of equipment used have been indicated.  
